# Direct Identification of Enteroviruses in Cerebrospinal Fluid of Patients with Suspected Meningitis by Nested PCR Amplification

**DOI:** 10.3390/v8010010

**Published:** 2016-01-06

**Authors:** Alexandr Krasota, Natalia Loginovskih, Olga Ivanova, Galina Lipskaya

**Affiliations:** 1 Belozersky Institute of Physico-Chemical Biology, Lomonosov Moscow State University, Moscow 119899, Russia; galina.lipskaya@gmail.com; 2 Chumakov Institute of Poliomyelitis and Viral Encephalitides, Moscow 142782, Russia; poliom@aha.ru; 3 Hygienic and Epidemiological Center in the Omsk Region, Omsk 644116, Russia; polioom@mail.ru

**Keywords:** Enterovirus, nested PCR, cerebrospinal fluids (CSF), direct genotyping, detection, identification, sequencing

## Abstract

Enteroviruses, the most common human viral pathogens worldwide, have been associated with serous meningitis, encephalitis, syndrome of acute flaccid paralysis, myocarditis and the onset of diabetes type 1. In the future, the rapid identification of the etiological agent would allow to adjust the therapy promptly and thereby improve the course of the disease and prognosis. We developed RT-nested PCR amplification of the genomic region coding viral structural protein VP1 for direct identification of enteroviruses in clinical specimens and compared it with the existing analogs. One-hundred-fifty-nine cerebrospinal fluids (CSF) from patients with suspected meningitis were studied. The amplification of VP1 genomic region using the new method was achieved for 86 (54.1%) patients compared with 75 (47.2%), 53 (33.3%) and 31 (19.5%) achieved with previously published methods. We identified 11 serotypes of the Enterovirus species B in 2012, including relatively rare echovirus 14 (E-14), E-15 and E-32, and eight serotypes of species B and 5 enteroviruses A71 (EV-A71) in 2013. The developed method can be useful for direct identification of enteroviruses in clinical material with the low virus loads such as CSF.

## 1. Introduction

The Enterovirus (EV) genus of the *Picornaviridae* family is divided into twelve species. Four human enterovirus species A, B, C, D comprises more than 100 serotypes. The EV genome, ribonucleic acid of positive polarity (+RNA), consists of approximately 7100–7500 base pairs. The virus-encoded RNA-dependent RNA polymerase does not possess proofreading property thereby endowing high mutation rate during replication [1]. In addition, Picornavirus genomes are subjected to frequent recombination events [2,3,4,5,6,7,8], adding to their plasticity and allowing rapid adaptation to therapeutic and/or immune pressure. EVs are the most common human viral pathogens [9] causing a broad range of illnesses including serous meningitis [10], encephalitis [10,11], syndrome of acute flaccid paralysis [12], myocarditis [13,14] and the onset of diabetes type 1 [15]. The EV infections of central nervous system (CNS) were associated with an increased risk of adult onset of schizophrenia or other psychosis [16]. In some cases, the CNS pathologies have detrimental impact on other organs as it was shown for fatal cases of cardiomyopathies caused by EV-A71 affected CNS [17].

With severe injuries of the CNS or heart tissue, the disease has the adverse prognosis; therefore, an accurate diagnosis is important for effective treatment. A routine medical practice of managing CNS and myocard viral pathologies with only standard symptomatic therapy is widely accepted, although antiviral and specific therapies could be used for improving the course and outcome of the disease. For the diseases with EV etiology, not only the virus detection but the virus identification and genotyping are important because distinct serotypes and their genogroups can differ in pathogenicity and virulence [18,19]. The efficacy of antiviral drugs in relation of certain serotypes and genogroups differs [20] therefore this information should be considered in the decision of using antiviral and specific therapies in the management of patients in future.

The precise intravital diagnostics of CNS can be established only by the detection of virus in CSF and subsequent virus serotyping. In many cases, the virus loads in such clinical specimens are quite low [21], therefore the amplification of genome region appropriate for serotyping requires highly sensitive PCR technics. The genome region coding enterovirus structural protein VP1 is commonly used for determination of serotype, genogroup and phylogenetic relationship of virus.

The aim of this work was to develop a highly sensitive method for rapid direct identification of enteroviruses by amplification of the whole genome region coding for viral structural protein VP1 and to compare it with the existing analogs.

The direct EV serotyping from CSF of patients with suspected meningitis was performed with different methods developed for direct investigation of clinical material, including nested PCR we have developed for amplification of the whole VP1 sequence.

The existing seminested PCRs for amplification the whole VP1 genomic region [22] or its part [23] and nested PCR for amplification of the part of VP1 [24] were used in this investigation for comparison.

## 2. Materials and Methods

### 2.1. Patients

CSF samples were collected from patients with suspected aseptic meningitis admitted to the hospitals in Omskaya oblast (West Siberia, Russia), within the framework of the national Public Health program “Non-polio Enterovirus Infection Surveillance and Prevention”. Collected specimens were delivered to Omsk virological laboratory and stored at −80 °C. The diagnosis of the suspected viral aseptic meningitis was based on clinical symptoms such as headache, fever, rigidness of neck, nausea/vomiting, photophobia, and negative CSF culture for bacterial test. Eighty-eight patients were admitted in 2012 and 71 in 2013. The retrospective direct serotyping of CSF samples was performed in Moscow in World Health Organisation Polio Regional Reference laboratory for Russian Federation and several New Independent States.

### 2.2. General Characteristic of Patients Included in the Study

In 2012, 88 patients were admitted (7 adults, mean age 23.6 years and 81 children, mean age 8.1 years). The male/female ratio was 1.3:1 and 1.4:1 in adults and children, respectively. The age range was from 18 to 32 years in adults and from 2 to 14 years in children. In 2013, 71 patients were admitted (9 adults, mean age 23.1 years and 62 children, mean age 6.5 years). The male/female ratio was 1.4:1 and 1.5:1 in adults and children, respectively. The age range was from 17 to 29 years in adults and from 1 to 14 years in children. The CSF samples were collected during the whole years.

### 2.3. RNA Extraction

DNase (1 µL) and 1 µL RNase A (Thermo Scientific EU, Vilnius, Lithuania) were added to 100 µL of each CSF sample and incubated at 37 °C for 30 min. For RNA extraction RNeasy^®^ Mini Kit (Qiagen, Hilden, Germany) was used according to the manufactures instructions. The RNA was eluted in 30 µL of H_2_O.

### 2.4. Reverse Transcription (RT)

Synthesis of complementary deoxyribonucleic acid (cDNA) was carried out in 20 µL reaction mixture containing 4 µL of 5× Super Script III First-Strand Buffer (Invitrogen, Carlsbad, CA, USA) 4 µL of 25 M hexamer primers, 2 µL of 0.1 M DTT, 0.5 µL of 20 mM nucleotide mixture, 100 U of Superscript III reverse transcriptase (Invitrogen, Carlsbad, CA, USA), 20 U of RiboLock RNase Inhibitor (Thermo Scientific EU, Vilnius, Lithuania) and 8.5 µL of the extracted RNA with 25 °C for 5 min, 50 °C for 60 min, and 95 °C for 5 min. 

### 2.5. Nested PCR

In the first-round PCR (total volume 50 µL), 5 µL of RT mixture was used (PCR 1) consisting of 24 µL of H_2_O, 5 µL of 10× Dream Taq PCR buffer (Thermo Scientific EU, Vilnius, Lithuania), 5 µL of each 10 µM 131 and 224 primers [25], (Table 1), 5 µL of 2 mM nucleotide mixture and 1 µL (5 ) of Dream Taq DNA polymerase (Thermo Scientific EU, Vilnius , Lithuania) using the following conditions: 95 °C for 5 min for denaturation, then 40 cycles of 30 s at 95 °C, 45 s at 42 °C, ramp 40%, and 1 min 50 s at 60 °C.

**Table 1:** Oligonucleotide primers used in this study and the resulted amplicons.

Primer	Position, nt	Resulted Amplicon, nt
224 ^d^	1977–1996 ^a^	1856
131 ^d^	3832–3812 ^a^	
HEVBS1695 ^e^	2375–2409 ^b^	1093
HEVBR132 ^e^	3467–3432 ^b^	
AK1	2298–2320 ^c^	1373
AK3	3670–3645 ^c^	

^a^ The positions are given relative to the genome of poliovirus 1, strain Mahoney; ^b^ The positions are given relative to the genome of echovirus 30, strain Bastiani; ^c^ The positions are given relative to the genome of enterovirus A71, strain BJ303; ^d^ Primers were described in [25]; ^e^ Primers were described in [22].

The second-round nested PCR (PCR 2) contained 2 µL of the first-round PCR, 27 µL of H2O, 5 µL of 10× Dream Taq PCR buffer (Thermo Scientific EU, Vilnius, Lithuania), 5 µL of each 10 µM HEVBS1695 and HEVBR132 primers [22] or 5 µL of each 10 µM primers for EV-A71 AK1: 5′-ATATGGTATCAGACNAAYTAYGT-3′ and AK3: 5′-AGGATACCACCGCARTCNCCNGGYTC-3′, 5 µL of 2 mM nucleotide mixture and 1 µL (5 U) of Dream Taq DNA polymerase (Thermo Scientific EU, Vilnius, Lithuania). PCR 2 was carried out under the following conditions: 95 °C for 2 min and then 40 cycles of 15 s at 95 °C, 30 s at 55 °C, 1 min 30 s at 68 °C and finally postcycling stage 10 min at 68 °C. 

Standard precautions were undertaken to avoid the risks of contamination. RNase free water as a negative control and E-9 RNA, cDNA and PCR1 product were used as positive controls for RT, PCR 1 and PCR 2 respectively. As additional methods, we used two seminested PCRs according to [22,23] and 1 nested PCR according to [24]. All amplifications were performed on Applied BiosystemsVerity amplificator (Singapore). PCR products were separated and visualized on 1.5% agarose gel stained with ethidium bromide and were purified using QIA quick^®^ gel extraction kit (Qiagen, Hilden, Germany) according to manufactures instructions.

### 2.6. Sequencing and Sequences Analysis

Sequencing was performed on ABI Prism 3130 genetic analyzer using Big Dye Terminator sequencing kit v. 3.1 (Applied Biosystems, Forester city, CA, USA). DNA Star TagMan program (DNA STAR, Madison, WI, USA) was used for analyzing raw sequence data. To determine the EV genotype, we compared the sequence homology between the amplified PCR products and sequences available in GenBank. The obtained sequences were assigned as the serotypes which gave the highest identity score in BLAST program [26].

### 2.7. Assay Sensitivity

The sensitivity relative to the results of cell culture infectivity of E-6 enterovirus serotype strain 52,725 was determined. The RNA was extracted from 100 µL of tenfold virus dilutions from 10^5^ to 10^−2^ cell culture infectious dose 50% end-point units (CCID_50_) in negative CSFs with RNeasy^®^ Mini Kit (Qiagen, USA) according to the manufactures instructions. RNAs representing from 10^4^ to 10^−3^ CCID_50_ per 5 µL were tested with VP1 RT-nested PCR amplification. Super Script III reverse transcriptase was used in RT reaction according to manufactures instructions.

The sequences of EVs identified in our assay were deposited in GeneBank sequence database, accession nos KU133569–KU133654.

## 3. Results

### 3.1. Comparative Analysis of the Existing and New Amplification Methods

**Figure 1:**
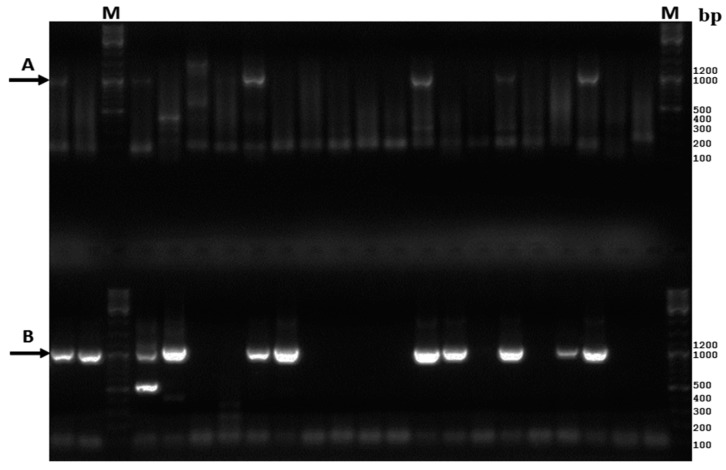
Gel electrophoresis of amplified CSF samples according to [22] above (**A**) and according to our method ant the bottom; (**B**) M-molecular weight marker. Black arrows indicate the bands which correspond to calculated weight of the amplicons.

One-hundred-fifty-nine CSF samples from patients with suspected serous meningitis were studied for enteroviruses. We evaluated one nested PCR and two seminested PCRs previously described in the literature that amplified full or a part of the genomic region coding for the viral structural protein VP1 [22,23,24]. Nested PCR amplification according to Casas I. *et al.* [24] resulted in the appearance of non-specific bands in gels and the PCR product of expected molecular weight was generated in 31 samples (19.5%). Seminested PCR according to Nix W.A. *et al.* [23], except the using of FastStart Taq DNA polymerase in the second round of PCR, detected EVs in 75 (47.2%) of CSF samples. The seminested PCR have been developed for amplification of the whole VP1 sequence for enteroviruses species B [22]. Using this method, we obtained the PCR product of correct molecular weight (Figure 1A) from 53 (33.3%) CSF samples.

Seminested PCR according to [23] was highly sensitive but amplified only ~350 to 400 of the VP1 genomic region and seminested PCR according to [22] missed 22 positive samples. Nested PCR [24] designed to amplify the part of the VP1 sequence showed low sensitivity compared with other tested methods.

As we needed a highly sensitive method to amplify the whole VP1 region and none of the tested methods was appropriate for our purpose, the new nested PCR allowing this was developed. In order to reach maximum sensitivity and specificity, we used highly degenerate primers 224 and 131 [25] with highly degenerate conditions for the first round of PCR and CODEHOP (Consensus-Degenerate Hybrid Oligonucleotide Primer) nested primers HEVBS1694-HEVBR132 and AK1-AK3 with adjusted conditions for the second round. The CODEHOP technology is a powerful tool for primer design which ensures high sensitivity and at the same time high specificity of amplification. They consist of 5′ consensus and 3′ degenerate parts and this approach provides increased sensitivity and the ability to amplify different serotypes/variants of the virus [27]. The primers HEVBS1694-HEVBR132 and AK1-AK3 amplify the whole VP1 genome region. Using this nested PCR, we identified EVs from 86 CSF samples (54.1%) including almost all samples that were positive in other methods. Also the amount of PCR products of the predicted molecular weight increased significantly compared with method [22] which also allowed obtaining of the full VP1 sequence (Figure 1B). The inner primers AK1 and AK3 were used to amplify VP1 genome region of EV-A71. These primers were designed to conservative sequences of VP3 and 2A genome regions of EV-A71 with the use of CODEHOP technology. AK1, AK3 primers allow to obtain PCR products of CV-B5 and E-18 and the resulting amplicon is longer than the PCR product of HEVBS1695 and HEVBR132 primers thereby we compared sequences flanked with AK1-AK3 primers with the database of GeneBank.

### 3.2. Assay Sensitivity

The VP1 RT nested PCR assay detected RNA extracted from 10^−2^ CCID_50_ per 5 µL of E-6 virus indicating that the assay is at least 100 fold more sensitive than cell culture for this strain of virus since 1 CCID_50_ defines the cell culture end point.

### 3.3. Retrospective Direct Genotyping of Enteroviruses Detected in CSF of Patients Hospitalized in 2012

Eighty-eight patients with suspected meningitis were admitted to the hospitals in Omskaya oblast in 2012 and were subjected to lumbal puncture. RNAs extracted from CSFs were amplified with our new nested PCR method and the amplicons of predicted molecular weight were obtained for 47 (53.4%) samples. Successful serotyping was achieved for all 47 positive samples. The serotype was assigned to each sample as the type which gave the maximal score by the means of BLAST program according to the comparison of obtained sequence with all sequences in the database of GeneBank. 11 serotypes of species B were identified in CSFs in 2012 including relatively rare E-14, E-15 and E-32 (Table 2).

**Table 2:** Direct serotyping of enteroviruses detected in cerebrospinal fluids (CSFs) of patients with serous meningitis admitted in 2012 and 2013.

2012				2013			
No.	CSF Specimen	EV Serotype	Accession No.	No.	CSF Specimen	EV Serotype	Accession No.
1	257-CSF/RUS/Omsk/2012	E-6	KU133571	1	376-CSF/RUS/Omsk/2013	E-11	KU133616
2	259-CSF/RUS/Omsk/2012	E-6	KU133573	2	377-CSF/RUS/Omsk/2013	E-11	KU133617
3	266-CSF/RUS/Omsk/2012	E-6	KU133577	3	380-CSF/RUS/Omsk/2013	E-11	KU133618
4	268-CSF/RUS/Omsk/2012	E-6	KU133579	4	381-CSF/RUS/Omsk/2013	E-11	KU133619
5	271-CSF/RUS/Omsk/2012	E-6	KU133581	5	394-CSF/RUS/Omsk/2013	E-11	KU133626
6	272-CSF/RUS/Omsk/2012	E-6	KU133582	6	395-CSF/RUS/Omsk/2013	E-11	KU133627
7	274-CSF/RUS/Omsk/2012	E-6	KU133583	7	400-CSF/RUS/Omsk/2013	E-11	KU133629
8	320-CSF/RUS/Omsk/2012	E-6	KU133602	8	402-CSF/RUS/Omsk/2013	E-11	KU133630
9	321-CSF/RUS/Omsk/2012	E-6	KU133603	9	404-CSF/RUS/Omsk/2013	E-11	KU133632
10	322-CSF/RUS/Omsk/2012	E-6	KU133604	10	412-CSF/RUS/Omsk/2013	E-11	KU133637
11	324-CSF/RUS/Omsk/2012	E-6	KU133605	11	419-CSF/RUS/Omsk/2013	E-11	KU133639
12	329-CSF/RUS/Omsk/2012	E-6	KU133608	12	430-CSF/RUS/Omsk/2013	E-11	KU133643
13	332-CSF/RUS/Omsk/2012	E-6	KU133610	13	443-CSF/RUS/Omsk/2013	E-11	KU133647
14	338-CSF/RUS/Omsk/2012	E-6	KU133612	14	445-CSF/RUS/Omsk/2013	E-11	KU133648
15	340-CSF/RUS/Omsk/2012	E-6	KU133613	15	446-CSF/RUS/Omsk/2013	E-11	KU133649
16	341-CSF/RUS/Omsk/2012	E-6	KU133614	16	448-CSF/RUS/Omsk/2013	E-11	KU133650
17	261-CSF/RUS/Omsk/2012	CV-A9	KU133574	17	452-CSF/RUS/Omsk/2013	E-11	KU133653
18	263-CSF/RUS/Omsk/2012	CV-A9	KU133575	18	391-CSF/RUS/Omsk/2013	EV-A71	KU133624
19	270-CSF/RUS/Omsk/2012	CV-A9	KU133580	19	434-CSF/RUS/Omsk/2013	EV-A71	KU133644
20	296-CSF/RUS/Omsk/2012	CV-A9	KU133590	20	437-CSF/RUS/Omsk/2013	EV-A71	KU133645
21	297-CSF/RUS/Omsk/2012	CV-A9	KU133591	21	450-CSF/RUS/Omsk/2013	EV-A71	KU133651
22	299-CSF/RUS/Omsk/2012	CV-A9	KU133593	22	451-CSF/RUS/Omsk/2013	EV-A71	KU133652
23	302-CSF/RUS/Omsk/2012	CV-A9	KU133594	23	382-CSF/RUS/Omsk/2013	CV-A9	KU133620
24	309-CSF/RUS/Omsk/2012	CV-A9	KU133597	24	393-CSF/RUS/Omsk/2013	CV-A9	KU133625
25	312-CSF/RUS/Omsk/2012	CV-A9	KU133599	25	408-CSF/RUS/Omsk/2013	CV-A9	KU133635
26	313-CSF/RUS/Omsk/2012	CV-A9	KU133600	26	409-CSF/RUS/Omsk/2013	CV-A9	KU133636
27	325-CSF/RUS/Omsk/2012	CV-A9	KU133606	27	386-CSF/RUS/Omsk/2013	E-7	KU133622
28	256-CSF/RUS/Omsk/2012	E-9	KU133570	28	413-CSF/RUS/Omsk/2013	E-7	KU133638
29	267-CSF/RUS/Omsk/2012	E-9	KU133578	29	428-CSF/RUS/Omsk/2013	E-7	KU133641
30	287-CSF/RUS/Omsk/2012	E-9	KU133589	30	403-CSF/RUS/Omsk/2013	E-30	KU133631
31	307-CSF/RUS/Omsk/2012	E-9	KU133596	31	405-CSF/RUS/Omsk/2013	E-30	KU133633
32	342-CSF/RUS/Omsk/2012	E-9	KU133615	32	455-CSF/RUS/Omsk/2013	E-30	KU133654
33	258-CSF/RUS/Omsk/2012	CV-B1	KU133572	33	390-CSF/RUS/Omsk/2013	CV-B2	KU133623
34	277-CSF/RUS/Omsk/2012	CV-B1	KU133584	34	407-CSF/RUS/Omsk/2013	CV-B2	KU133634
35	298-CSF/RUS/Omsk/2012	CV-B1	KU133592	35	425-CSF/RUS/Omsk/2013	CV-B2	KU133640
36	265-CSF/RUS/Omsk/2012	CV-B1	KU133576	36	383-CSF/RUS/Omsk/2013	E-18	KU133621
37	328-CSF/RUS/Omsk/2012	CV-B1	KU133607	37	399-CSF/RUS/Omsk/2013	E-18	KU133628
38	279-CSF/RUS/Omsk/2012	E-11	KU133585	38	429-CSF/RUS/Omsk/2013	CV-B4	KU133642
39	282-CSF/RUS/Omsk/2012	E-11	KU133586	39	439-CSF/RUS/Omsk/2013	CV-B5	KU133646
40	318-CSF/RUS/Omsk/2012	E-11	KU133601	–	–	–	–
41	284-CSF/RUS/Omsk/2012	E-18	KU133587	–	–	–	–
42	330-CSF/RUS/Omsk/2012	E-18	KU133609	–	–	–	–
43	253-CSF/RUS/Omsk/2012	E-32	KU133569	–	–	–	–
44	286-CSF/RUS/Omsk/2012	E-30	KU133588	–	–	–	–
45	304-CSF/RUS/Omsk/2012	E-14	KU133595	–	–	–	–
46	310-CSF/RUS/Omsk/2012	CV-B2	KU133598	–	–	–	–
47	333-CSF/RUS/Omsk/2012	E-15	KU133611	–	–	–	–

### 3.4. Retrospective Direct Genotyping of Enteroviruses Detected in CSFs of Patients Hospitalized in 2013

Seventy-one patients with suspected meningitis were admitted to the hospitals in Omskaya oblast in 2013. Enteroviruses were detected in 39 (54.9%) CSF specimens. Successful genotyping was achieved for all 39 positive samples. Eight serotypes of species B and 5 EV-A71 (Table 2) were identified in CSFs in 2013 and the phylogenetic analysis displayed that EV A71 from Omskaya oblast was related to EV A71 from China identified in 2008.

To avoid omission of the serotypes of species A (except EV-A71), species C, and species D, we used the method described by Nix *et al.* [23] or primers AN89 and AN88 for the second round of PCR, but none of these serotypes was detected.

The spectrum of EVs in 2012 changed in 2013 (Table 3) and these data conform to previous investigations [28]. The serotype E-6 was prevailing in 2012 and E-11 became prevailing in 2013.

**Table 3:** Number of enterovirus serotypes identified in patients with meningitis through direct serotyping in CSFs.

Identified Serotype	No. and % (in Parentheses) of EV Serotype to All EVs Identified during the Corresponding Year	
	2012	2013
Coxsackievirus A9	11 (23.4)	4 (10.3)
Coxsackievirus B1	5 (10.6)	0
Coxsackievirus B2	1 (2.1)	3 (7.7)
Coxsackievirus B4	0	1 (2.6)
Coxsackievirus B5	0	1 (2.6)
Echovirus 6	16 (34.1)	0
Echovirus 7	0	3 (7.7)
Echovirus 9	5 (10.7)	0
Echovirus 11	3 (6.4)	17 (43.5)
Echovirus 14	1 (2.1)	0
Echovirus 15	1 (2.1)	0
Echovirus 18	2 (4.3)	2 (5.1)
Echovirus 30	1 (2.1)	3 (7.7)
Echovirus 32	1 (2.1)	0
Enterovirus A71	0	5 (12.8)

## 4. Discussion

According to the currently existing epidemiological data the importance of non-polio enteroviruses (NPEVs) as the etiological agents of severe pathologies of CNS, heart, and pancreas rise considerably. It may be linked to the results of the Global Polio Eradication Initiative of World Health Organization (WHO) a great decrease of the Poliomyelitis morbidity and circulation of polio viruses, a replacement of the oral polio vaccine (OPV) by inactivated polio vaccine in many countries [29,30]. To date, viruses of serotypes belonging to species B and EV71 are some of the major etiological agents of severe neurological diseases and cardiomyopathies in developed countries [19,31,32]. For instance, EV-A71 and some coxsackieviruses are able to cause poliomyelitis-like acute flaccid paralysis [30,33]. Based on the importance of these viruses we focused our investigation on them.

Several molecular serotyping methods for amplification of the whole EV VP1 genomic region or its part directly from clinical material were developed [22,23,24,31,34,35,36,37,38].

Evaluation of published highly sensitive methods developed for direct enterovirus serotyping showed that the seminested PCR described in [23] had the highest effectiveness for serotyping. The non-specific amplification observed with the method described in [24] may be due to the usage of degenerate primers in the second round of PCR.

The method described in this paper allowed us to identify the majority of EVs in the CSF samples. This technology also allowed amplifying the whole VP1 genomic region of the identified EVs, what is preferable for genotyping and phylogenetic purposes. We used species-specific primers for species B and serotype specific for EV-A71, and this perhaps ensued achievement of maximal sensitivity among tested methods. At the same time, it caused some disadvantages as it requires the usage of other primers in the second round of PCR in order to amplify the remainder of EV serotypes. This problem can be resolved by amplification of the VP1 gene in the second round of PCR with primers specific to EV species A, C, D. For detection of possibly missed EVs of these species, the method by Nix W.A. *et al.* [23] can be used. In our investigation, we have not revealed EVs which do not belong to species B or serotype EV71 with methods based on generic primers for the second round of PCR.

The ability to establish persistent infection is an intrinsic trait of EVs. One of the mechanisms for this phenomenon is the acquiring of deletions in the 5′ region of EV RNA [39]. The persisting virus can be associated with adverse effects for the host. For example, enteroviral persistence in myocardium has been associated with progressive cardiac dysfunction. On the contrary, patients who eliminated the virus showed substantial improvement of hemodynamic parameters [40]. The experiments in mice suggest that enterovirus coxsackievirus B3 (CV-B3) may persist in the CNS as a low-level, non-cytolytic infection, causing ongoing inflammatory lesions [41]. Taking into consideration that the enterovirus genome can lose the particular sequences of the 5′ non-coding genomic region during persistent infection, and this region has been widely used as a gold standard for EV detection [22,23], it appears that the amplification of VP1 sequence sequence could be more applicable for precise diagnostics of EV diseases. Besides, it provides more important information that can also furnish clues to virus transmission pathways during epidemiologic field investigations [23].

Considering the increasing role of NPEVs in the structure of infectious pathology, the development of anti-enteroviral vaccines and hyperimmune serums could be recommended. The vaccines against the most virulent serotypes and their genotypes can be developed and used based on epidemiological surveillance as, for instance, it is done for the prophylaxis of the influenza virus. The rapid accurate diagnosis could provide the timely correction of treatment using antiviral and specific drugs, thereby improving the course of the disease and prognosis. Highly sensitive and specific methods are required for precise diagnostics of enterovirus infections of CNS and heart. The method for direct serotyping of EVs described here allows quick determination of EV serotypes and provides information about the whole VP1 genomic region, and, therefore, could be useful in clinical, epidemiological surveillance and research practice.

## 5. Conclusions

Highly sensitive method for amplification of the genome region coding the whole entero virus structural protein VP1 was developed. It demonstrated to be effective for investigation of the cerebrospinal fluids (CSF)—the clinical material with quite low viral loads. This method can be useful in scientific, clinical and epidemiological research.
